# Is Fluorescence‐Guided Surgery Reliable for the Treatment of MRONJ? A Systematic Review and Meta‐Analysis

**DOI:** 10.1111/odi.70320

**Published:** 2026-03-31

**Authors:** Paolo De Angelis, Alessandro Donato Tescione, Ivan Michele Aniceto, Cosimo Rupe, Gioele Gioco, Romeo Patini, Carlo Lajolo

**Affiliations:** ^1^ Department of Head and Neck and Sense Organs, School of Dentistry Fondazione Policlinico Universitario Agostino Gemelli IRCCS, Università Cattolica del Sacro Cuore Rome Italy

**Keywords:** medication‐related osteonecrosis of the jaw, MRONJ, osteonecrosis, osteonecrosis of the jaw

## Abstract

**Background:**

Identification of healthy bone margins is a critical step in the surgical resective treatment of medication‐related osteonecrosis of the jaw (MRONJ). Fluorescence‐guided surgery (FGS) has been proposed as a method to identify resection margins and improve clinical outcomes. The purpose of this review was to systematically evaluate the success rate of FGS.

**Materials and Methods:**

The Cochrane Central Register, PubMed, Scopus and Web of Science databases were searched. Success was defined as the absence of exposed necrotic bone with full mucosal coverage and no signs of MRONJ recurrence, assessed at overall follow‐up. The risk of bias was evaluated using the Newcastle‐Ottawa Scale, the Moga Index and the GRADE approach. The PRISMA protocol was followed to evaluate and present the results.

**Results:**

Nine studies met the inclusion criteria, comprising a total of 285 patients. Of the 314 lesions treated with FGS, 285 achieved complete healing during a mean follow‐up of 13.0 months, with an overall success rate of 88.54%. The fluorescence modalities used included autofluorescence, tetracycline‐induced fluorescence and near‐infrared fluorescence imaging with indocyanine green.

**Conclusion:**

Fluorescence‐guided surgery appears to be a promising adjunctive tool for the surgical management of MRONJ, contributing to high success rates.

## 1 Introduction

Medication‐related osteonecrosis of the jaw (MRONJ) is a complex and often debilitating condition that has recently emerged as a significant focus of scientific research. Owing to the relative novelty of the disease, definitions and classification systems have been proposed between European (Bedogni et al. 2024) and American frameworks (Khosla et al. 2007) (Ruggiero et al. 2022).

According to the most recent SIPMO–SICMF Expert Panel, MRONJ is defined as ‘an adverse drug reaction characterised by the progressive destruction and death of bone that affects the mandible and maxilla of patients exposed to medications known to increase the risk of the disease, in the absence of previous radiation therapy’ (Bedogni et al. 2024). In contrast, the American Association of Oral and Maxillofacial Surgeons (AAOMS) defines MRONJ as the presence of exposed bone or bone that can be probed through an intraoral or extraoral fistula, in the maxillofacial region persisting for more than 8 weeks (Ruggiero et al. 2022).

MRONJ primarily occurs in patients undergoing long‐term treatment with antiresorptive agents such as bisphosphonates and denosumab. However, it has also been associated with biological therapies, including mammalian target of rapamycin inhibitors, tyrosine kinase inhibitors and anti‐vascular endothelial growth factor agents (Nicolatou‐Galitis et al. 2019).

Initially identified in 2003 as bisphosphonate‐related osteonecrosis of the jaw, the condition was renamed MRONJ in 2015 to encompass a broader spectrum of causative medications, including antiangiogenic agents (Ruggiero et al. 2014).

Clinically, MRONJ typically presents as exposed necrotic bone in the jaw, often accompanied by local symptoms such as pain, swelling and signs of infection. Diagnosis and staging rely on both clinical and radiographic findings. Early manifestations may include subtle sclerosis, whereas advanced stages often reveal bone fragmentation and sequestration. Radiological imaging can also assist in determining the extent of the disease (Hutchinson et al. 2010).

Medical or conservative therapy, including antimicrobial rinses and antibiotics, may be effective in the early stages, whereas surgical debridement or resection is often regarded as the most promising therapeutic approach.

The surgical management of MRONJ presents several challenges, including the risk of recurrence, as reported by Han‐Jin Ruan, who documented a recurrence rate of 16.85% with no significant difference between the maxilla and mandible (Ruan et al. 2024). A major intraoperative challenge lies in distinguishing healthy from necrotic bone, which is currently assessed by the presence or absence of bleeding and by bone colour. This method increases the likelihood of incomplete resection. Another contributing factor is the reduced bone turnover induced by antiresorptive therapy, which delays post‐surgical healing and leaves the surgical site more susceptible to reinfection (Mücke et al. 2011). Additionally, systemic conditions such as diabetes, poor nutritional status, smoking, poor oral hygiene and immunosuppression may adversely affect recovery.

To improve surgical precision, preoperative imaging techniques (e.g., cone‐beam CT), are valuable for surgical planning. However, the actual extent of MRONJ may not always be clearly delineated on radiographic images, and the clinician's intraoperative assessment remains crucial for defining resection margins based on parameters such as colour, bleeding and signs of infection.

Recently, fluorescence‐guided surgery (FGS) has been proposed to address the limitations of conventional surgical management of MRONJ and is emerging as a promising technique. Fluorescence may assist in differentiating necrotic bone from healthy tissue in real time by exploiting the differential fluorescence emitted by vital and necrotic bone, thereby enabling intraoperative visualisation of bone vitality and potentially reducing the risk of residual disease. Several fluorescence modalities have been described, including autofluorescence (Giovannacci et al. 2017), tetracycline‐induced fluorescence (Pautke et al. 2011), quantitative light‐induced fluorescence, which exploits bacterial biofluorescence (Hwang et al. 2025) and near‐infrared fluorescence imaging, which uses fluorophores such as indocyanine green (ICG) (Zhi et al. 2025; Desmettre et al. 2000).

Fluorescence imaging has emerged as a valuable adjunct in bone surgery, both in preclinical models (Ristow et al. 2020) and clinical studies (Otto et al. 2016), due to its potential to accurately delineate necrotic tissue, optimise the extent of bone resection and preserve healthy bone. These factors are essential for achieving mucosal healing and reducing disease recurrence in patients with MRONJ. Although fluorescence‐guided surgery appears promising, its precise prognostic impact on surgical outcomes remains to be fully elucidated.

Therefore, the aim of this systematic review was to evaluate the success rate of FGS in the treatment of MRONJ.

## 2 Materials and Methods

This review was registered in PROSPERO (CRD420251071308) and conducted in accordance with the Preferred Reporting Items for Systematic Reviews and Meta‐Analyses (PRISMA) 2020 statement (Page et al. 2021). The PRISMA checklist is provided in Appendix S1. The research question was formulated using the PICOT framework as follows:

- Population: Patients with MRONJ undergoing surgical treatment.
- Intervention: Fluorescence‐guided surgery for MRONJ.
- Comparison: Conventional surgery for MRONJ or absence of a comparator.
- Outcome: Success rate, defined as complete mucosal healing with no exposed necrotic bone, absence of pain or suppuration and no signs of MRONJ recurrence after surgical treatment.
- Time: Studies with a mean follow‐up period of at least 6 months after surgical treatment.

### 2.1 Eligibility Criteria

The inclusion criteria were as follows:

- Human studies.
- Randomised controlled clinical trials (RCTs), prospective or retrospective studies including at least 10 patients.
- Patients undergoing fluorescence‐guided bone surgery for MRONJ treatment.
- Studies reporting detailed information on the surgical approach used and providing sufficient data on clinical outcomes.
- A minimum mean follow‐up of 6 months.
- If multiple publications on the same patient cohort were available, only the study with the longest follow‐up and/or most comprehensive dataset was included.
- The study provided an explicit definition of MRONJ diagnosis and success criteria.

Studies not meeting the above criteria were excluded.

### 2.2 Search Strategy

A systematic search was conducted independently and in duplicate by two authors (P.D.A. and G.G.) using the following electronic databases: PubMed, Cochrane Library, Scopus and Web of Science.

The search strategy was designed to identify peer‐reviewed publications in English up to 25 June 2025 and is reported in Table S1.

A manual search was also performed independently and in duplicate by two authors (A.D.T. and I.M.A.) by screening the following journals: Journal of Oral and Maxillofacial Surgery, International Journal of Oral and Maxillofacial Surgery, Journal of Oral Pathology and Medicine, Journal of Cranio‐Maxillofacial Surgery, Oral Oncology, Oral Surgery, Oral Medicine, Oral Pathology and Oral Radiology, Oral Diseases, Clinical Oral Investigations and Journal of Dentistry.

### 2.3 Study Selection and Data Collection

Title and abstract screening were conducted independently by two review authors (P.D.A. and G.G.). Subsequently, a comprehensive full‐text analysis was performed for all studies considered suitable for inclusion. Data were extracted using a specifically designed Microsoft Excel spreadsheet (Microsoft Corp., Redmond, WA, USA). All steps were carried out independently and in duplicate by the two authors and in cases of disagreement, a third senior author (R.P.) was consulted. The inter‐reviewer reliability (Cohen's kappa) for title and abstract screening as well as full‐text analysis was calculated.

Only studies demonstrating adequate clinical homogeneity in terms of population, intervention, outcomes and comparable follow‐up duration were included in the meta‐analysis. Studies that did not meet these criteria were analyzed qualitatively.

Data on the characteristics of the included studies (i.e., general study information, number of participants, demographic characteristics, study design, year, diagnostic criteria, follow‐up duration, overall success rate and success rate at 6 and 12 months) and data related to MRONJ treatment (MRONJ stage, surgical protocol and type of fluorescence) were collected and entered into a customised data collection form independently by three authors (P.D.A., A.D.T. and G.G.).

If multiple follow‐up assessments were available, the latest follow‐up was used for the primary analysis. Re‐interventions due to MRONJ recurrence were counted as treatment failures from the first surgery. Furthermore, subgroup analyses at 6‐ and 12‐month follow‐up intervals were performed (Bedogni et al. 2024).

### 2.4 Risk of Bias Assessment

The quality of each included study was evaluated independently and in duplicate by two reviewers (P.D.A. and A.D.T.), and conclusions were reached through discussion. In cases of disagreement during quality assessment, a third investigator (C.L.) reviewed the study, and consensus was achieved through discussion. For studies that included both a test group (fluorescence‐guided surgery) and a control group, methodological quality was assessed using the Newcastle–Ottawa Scale (Stang 2010). Single‐arm clinical studies evaluating the efficacy of fluorescence‐guided surgery were assessed using the Institute of Health Economics checklist developed by Moga et al. (2012), which is recognised for appraising case series in systematic reviews. Studies receiving between 9 and 7 stars on the Newcastle–Ottawa Scale were considered to have a low risk of bias; those scoring between 4 and 6 stars were assessed as having a moderate risk, while studies with fewer than 4 stars were defined as having a high risk of bias. The remaining studies were evaluated using the Moga et al. tool and classified according to the percentage of applicable criteria answered ‘Yes.’ Risk of bias was categorised as low if the total score ranged from 17 to 20/20, moderate if between 13 and 16/20 and high if below 13/20.

The certainty of the evidence was evaluated following the GRADE approach.

### 2.5 Publication Bias

Owing to the small number of studies included in this meta‐analysis, a formal assessment of publication bias (e.g., Egger's test, trim‐and‐fill method) was not conducted, in accordance with current recommendations, as such tests lack sufficient power and may produce misleading results when fewer than 10 studies are available. Although a funnel plot was visually inspected, its interpretation should be approached with extreme caution.

### 2.6 Data Synthesis

Proportions were pooled using the metaprop() function from the meta package in R.

This function was used to perform meta‐analyses of single proportions by calculating an overall pooled estimate from studies reporting a single‐proportion outcome.

Because proportions often display variance instability, particularly when approaching 0 or 1, the logit transformation (sm = ‘PLOGIT’) was applied. This approach is especially appropriate in the present context, where observed proportions were relatively high (ranging from 0.77 to 1.00) and the number of included studies was limited. Back‐transformed results were presented for ease of interpretation. Success was defined as the absence of exposed necrotic bone with full mucosal coverage and no signs of MRONJ recurrence. This was investigated at the overall follow‐up and, when available, at 6 and 12 months.

An alpha significance level of 0.05 was applied in all analyses. Statistical analyses were performed using RStudio software, version 4.3.1 (RStudio, PBC, Boston, MA, USA).

## 3 Results

### 3.1 Study Selection

The electronic search initially identified 123 articles and the hand search yielded 2 additional studies. After duplicate removal, 83 articles were screened. Of these, 56 were excluded after a careful review of titles and abstracts. The full texts of 27 potentially eligible articles were subsequently assessed, and 17 were excluded. Ultimately, 9 studies were included in this review (Zhi et al. 2025; Assaf et al. 2014; Otto et al. 2016, 2021; Ristow et al. 2017; Giudice et al. 2018; Giovannacci et al. 2025; Aljohani et al. 2018; Hauer et al. 2020). The search process and the reasons for exclusion are illustrated in the PRISMA flow chart (Figure 1). The inter‐reviewer reliability (Cohen's kappa) for title and abstract screening as well as full‐text analysis was 0.94, indicating almost perfect agreement between the two examiners.

**FIGURE 1:**
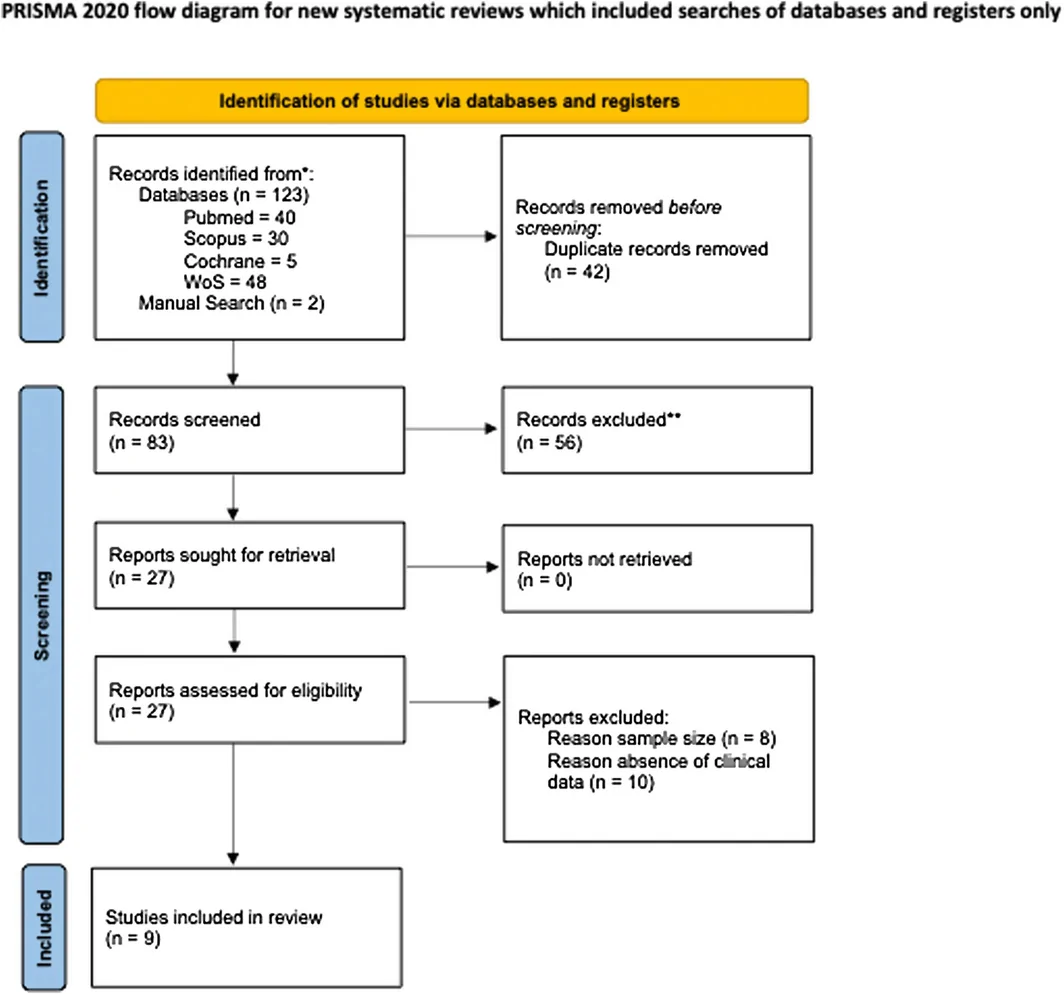
PRISMA flow diagram illustrating the study selection process.

### 3.2 Study Characteristics

The characteristics of the included studies (i.e., including general study information, number of participants, demographic data, study design, diagnostic criteria) and specific information such as the number of MRONJ lesions, overall success rate and success rates at 6 and 12 months are presented in Table 1.

**TABLE 1:** Characteristics of the included studies.

Author and year	Study design	Fluorescence technique	Test group		Control group		Success rate		Mean follow‐up (Months)	Site		Stage				Drug exposure			Primary disease	
			Sub.	Lesions	Sub.	Lesions	Test	Control	(Months)	Upper jaw	Lower jaw	0	I	II	III	BP	Dmab	BP+ Dmab	Ost	Onc
Assaf et al. (2014)	P	TF	20	20	—	—	20/20 (100%)	—	12	8	15	0	2	9	9	20	—	—	2	18
Otto et al. (2016)	P	TF	54	65	—	—	56/65 (86.2%)	—	12.9	25	40	1	14	42	8	47	3	4	9	45
Ristow et al. (2017)	RCT	TF vs. AF	34 (18 TF/16 AF)	41 (26 TF/25 AF)	—	—	38/41 (92.7%) 20/22 TF 18/19 AF	—	12	18 (7 TF/11 AF)	33 (19 TF/14 AF)	0	4 (3 TF/1 AF)	41 (20 TF/21 AF)	6 (3 TF/3 AF)	32	—	8	6	34
Giudice et al. (2018)	RCT	AF	18	16	15	17	15/16 (93.8%)	15/17 (88.2%)	12	12 (5 AF/5 Non‐AF)	29 (14 AF/15 Non‐AF)	0	6	6	7	16	2	—	8	10
Hauer et al. (2020)	R	TF	26	32	—	—	32/32 (100%)	—	20.5	10	22	0	3	24	5	18	4	4	26	0
Otto et al. (2021)	R	AF	75	82	—	—	67/82 (81.7%)	—	12	31	51	3	3	62	14	53	15	7	10	65
Zhi et al. (2025)	P	ICG–NIR	14	14	54	54	12/14 (85.71%)	39/54 (72.22%)	6	22 (6 NIR/16 Non‐NIR)	46 (8 NIR/38 Non‐NIR)	0	0	9	5	14	—	—	1	13
Aljohani et al. (2018)	R	TF	22	22	31	31	17/22 (77.3%)	21/31 (67.7%)	10	23	46	3	nd	nd	nd	32	31	—	nd	nd
Giovannacci et al. (2025)	P	AF	22	22	—	—	21/22 (95%)	—	18.3	4	18	0	8	8	6	13	3	6	9	13

Abbreviations: AF, autofluorescence; BP, bisphosphonates; Dmab, denosumab; FU, follow‐up; ICG–NIR, indocyanine green near‐infrared imaging; L, lesions; nd, not defined; Onc, onco‐haematological malignancy; Ost, osteoporosis; P, prospective; R, retrospective; RCT, randomised controlled trial; Sub., number of included subjects; TF, tetracycline‐induced fluorescence.

Among the included studies, two were RCTs (Otto et al. 2016; Ristow et al. 2017), four were prospective clinical studies (Zhi et al. 2025; Assaf et al. 2014; Otto et al. 2016; Giovannacci et al. 2025), and three were retrospective clinical studies (Otto et al. 2021; Aljohani et al. 2018; Hauer et al. 2020). With regard to the fluorescence modality, four studies employed native autofluorescence (AF) (Ristow et al. 2017; Giudice et al. 2018; Otto et al. 2021; Giovannacci et al. 2025), five used tetracycline‐induced fluorescence (TF) following short‐term antibiotic labelling (Assaf et al. 2014; Otto et al. 2016; Ristow et al. 2017; Aljohani et al. 2018; Hauer et al. 2020) and one adopted indocyanine‐green near‐infrared imaging (ICG‐NIR) (Zhi et al. 2025). Consequently, the VELscope device was used for fluorescence detection in eight studies, while a dedicated NIR camera was employed in the study using ICG (Zhi et al. 2025). Three studies included a control group treated without a fluorescence‐ guided approach (Zhi et al. 2025; Giudice et al. 2018; Aljohani et al. 2018). Follow‐up durations ranged from 3 to 36 months, with only two studies reporting follow‐up periods longer than 1 year (Otto et al. 2016, 2021). Across all studies, MRONJ stage II was the most prevalent, as classified according to AAOMS criteria.

All therapeutic protocols included both systemic antibiotic prophylaxis and systemic antibiotic therapy; however, significant differences were observed in the type of antibiotic administered, as well as in its timing and dosage. Tetracyclines, penicillins and metronidazole were the most frequently used agents, administered either individually or in combination. All authors prescribed chlorhexidine mouth rinses (0.12%–0.20%), and two studies incorporated Nd:YAG low‐level laser therapy (Assaf et al. 2014; Otto et al. 2016). Bone removal was generally performed using conventional rotary instruments, with the only alternative being an Er:YAG laser‐based approach described by Giovannacci et al. (2025).

In two studies, the reported success rate included not only primary healing but also outcomes following surgical reintervention. Giovannacci et al. (2025) observed an initial success rate of 95% after the first procedure, which increased to 100% after reintervention in one patient with incomplete healing (Giovannacci et al. 2025). Similarly, Otto et al. (2016) reported an improvement in the success rate from 86.2% after initial treatment to 95.4% following additional procedures during the observation period. In both studies, reinterventions were consistently performed using the same fluorescence‐guided surgical approach as the initial one, with no transition to conventional techniques or alternative methods.

Bisphosphonates were administered in all nine included studies and represented the most frequently reported antiresorptive therapy. Exposure to denosumab was documented in six studies (Otto et al. 2016, 2021; Giudice et al. 2018; Hauer et al. 2020; Aljohani et al. 2018; Giovannacci et al. 2025), whereas no denosumab use was reported in three studies (Assaf et al. 2014; Ristow et al. 2017; Zhi et al. 2025). Combined exposure to bisphosphonates and denosumab was observed in five studies (Otto et al. 2016, 2021; Ristow et al. 2017; Hauer et al. 2020; Giovannacci et al. 2025). A detailed overview of the patients' medical history and drug exposure is presented in Table 1.

Patient‐reported outcome measures, such as postoperative pain, oral function and quality of life, were rarely evaluated. Giudice et al. reported significantly lower visual analogue scale scores in the autofluorescence group compared with the control group during the first three postoperative days (Giudice et al. 2018). Similarly, Giovannacci et al. observed low pain levels on the first postoperative day, with no patients requiring major analgesic therapy and a rapid recovery of oral function (Giovannacci et al. 2025).

### 3.3 Synthesis of Results

Across the 9 included studies, a total of 285 patients with MRONJ, corresponding to 314 lesions, underwent fluorescence‐guided surgical treatment. Among these, 278 MRONJ lesions achieved complete healing during a mean follow‐up of 13.0 months, resulting in an overall success rate of 88.54%.

Among MRONJ‐affected sites, 161 lesions were treated using TF, achieving a reported success rate of 90.06%; 139 lesions were treated using AF with a success rate of 87.05%; and 14 were treated using ICG‐NIR, achieving a success rate of 85.71%.

Of the included studies, only three incorporated a control group, comprising a total of 100 control patients. Across 102 control lesions, a success rate of 73.53% was observed following conventional surgery.

A subgroup analysis based on follow‐up duration was also conducted at 6 and 12 months after surgery.

At the 6‐month time point, 4 studies reported clinical outcomes, with 88 out of 99 treated lesions healed, corresponding to a 6‐month success rate of 88.77%.

At the 12‐month time point, only 2 studies provided data, reporting 52 healed lesions out of 56, corresponding to a 12‐month success rate of 92.98%.

### 3.4 Results of Meta‐Analysis

Among the included studies, the meta‐analysis was performed on 6 studies (Assaf et al. 2014; Otto et al. 2016, 2021; Ristow et al. 2017; Giudice et al. 2018; Giovannacci et al. 2025).

The quantification of heterogeneity showed tau^2^ = 0, tau = 0 and *I* ^2^ = 0.0% [0.0%; 70.8%], indicating no observed heterogeneity among studies.

The tests for heterogeneity further supported this finding: the Wald test yielded Q = 1.79 with 6 degrees of freedom and *p* = 0.9381, while the likelihood ratio test produced Q = 4.98 with 6 degrees of freedom and *p* = 0.5465.

Both the common‐effect and random‐effects models estimated an overall pooled proportion of approximately 0.935, with a 95% confidence interval [0.8965; 0.9598]. The results are illustrated in the forest plot (Figure 2).

**FIGURE 2:**
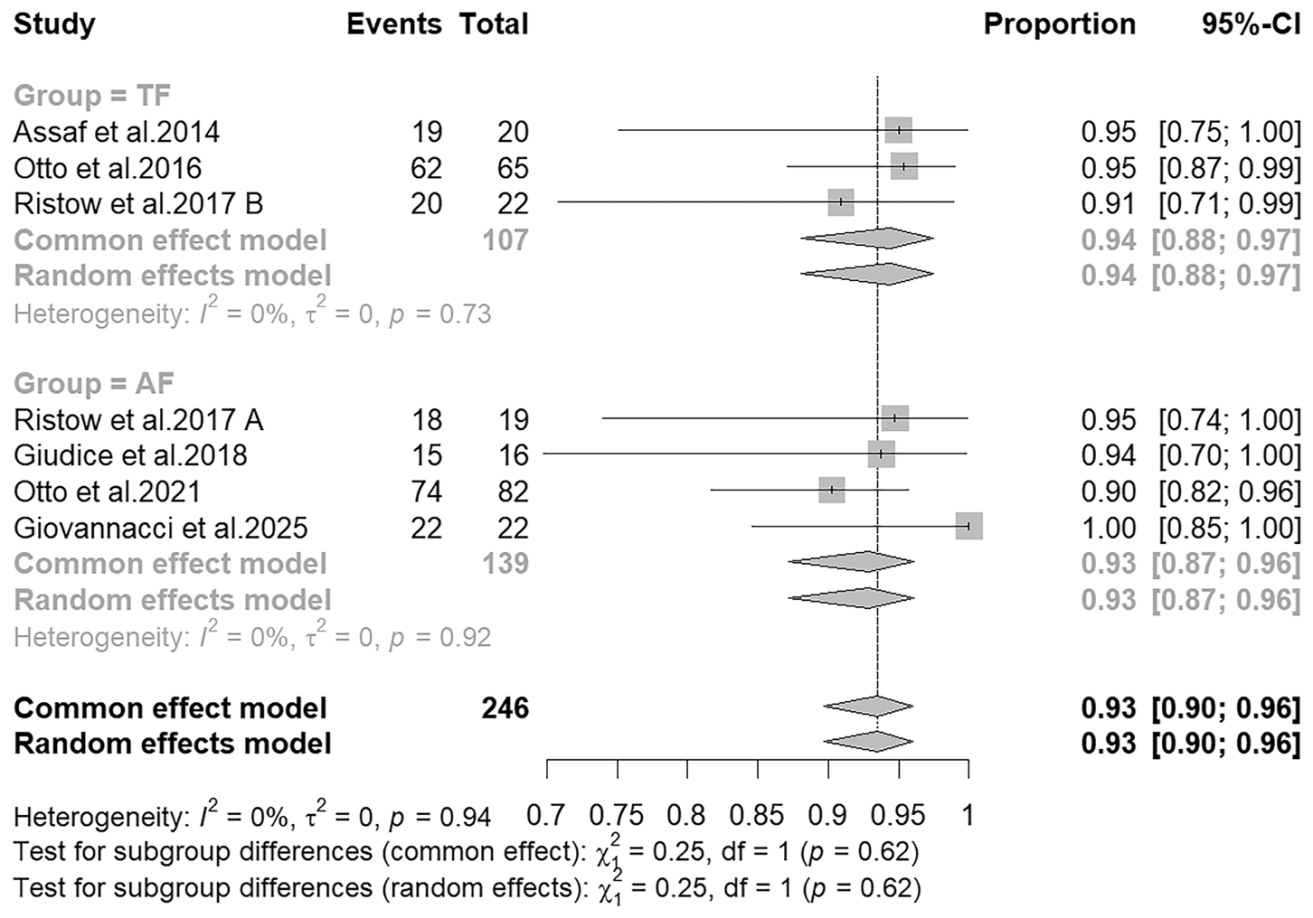
Forest plot showing pooled success rates of fluorescence‐guided surgery for MRONJ treatment.

Furthermore, a subgroup meta‐analysis was performed according to the treatment approach (TF versus AF). The test for subgroup differences was not significant (Q = 0.25, d.f. = 1, *p* = 0.6177), indicating that the pooled proportions did not differ significantly between the TF and AF subgroups. The results are presented in the forest plot (Figure 2).

### 3.5 Risk of Bias Assessment and Evidence Certainty

The risk of bias in comparative studies was assessed using the Newcastle‐Ottawa Scale and is reported in Table 2. Two RCTs, Ristow et al. (2017) and Giudice et al. (2018), were considered to have a low risk of bias, obtaining scores of 9/9 and 8/9, respectively. Two observational studies, Zhi et al. (2025) and Giovannacci et al. (2025), received scores of 6/9, as they did not obtain any stars in the comparability and outcome domains.

**TABLE 2:** Risk of bias assessed using the Newcastle‐Ottawa Scale.

Study	Selection (★)	Comparability (★)	Outcome (★)	Total score
Giudice et al. (2018)	★★★★	★★	★★★	9/9
Ristow et al. (2017)	★★★★	★★	★★	8/9
Aljohani et al. (2018)	★★★★	—	★★	6/9
Zhi et al. (2025)	★★★★	—	★★	6/9

*Note:* ★, star assigned per Newcastle–Ottawa Scale criteria (maximum score = 9).

The risk of bias assessment for non‐comparative studies, conducted using the Moga et al. (2012) scale, is presented in Table 3. Assaf et al. (2014), Otto et al. (2016), Hauer et al. (2020) and Giovannacci et al. (2025) achieved scores indicative of a low risk of bias. Specifically, Otto et al. (2016) scored 95%, Assaf et al. (2014) scored 90% and both Giovannacci et al. (2025) and Hauer et al. (2020) scored 85%, while Otto et al. (2021) obtained a score of 80%.

**TABLE 3:** Risk of bias assessed using the Moga et al. (2012) scale.

Assaf et al. (2014)	Low risk 18/20 (90%)
Otto et al. (2016)	Low risk 19/20 (95%)
Hauer et al. (2020)	Low risk 17/20 (85%)
Otto et al. (2021)	Moderate risk 16/20 (80%)
Giovannacci et al. (2025)	Low risk 17/20 (85%)

For the outcome ‘success rate after at least a mean of one year of follow‐up,’ the certainty of evidence was rated as moderate, with a very low strength of recommendation. The GRADE assessment is reported in Table 4.

**TABLE 4:** GRADE assessment of the certainty and strength of evidence.

Outcome	Number of studies	Study design	Result (Proportion)	95% confidence interval	Certainty (GRADE)	Strength of recommendation	Notes
Overall success rate	6	Randomised trials and observational studies	AF: 94% TF: 93%	AF: 88%–97% TF: 87%–96%	ΟΟΟΟ Very low	↑ (Conditional)	Imprecision and indirectness of evidence

### 3.6 Funnel Plot

The funnel plot appeared fairly symmetrical around the vertical line, suggesting the absence of evident publication bias. The studies were distributed on both sides of the central estimate without any clear clustering, further indicating that no strong bias was present. However, given the limited number of studies, the interpretation of the funnel plot should be approached with caution, as it is difficult to reliably assess publication bias when only a few studies are available. The funnel plot is presented in Figure 3. In summary, the funnel plot did not reveal clear evidence of publication bias; nonetheless, its interpretation should be made cautiously due to the relatively small number of included studies.

**FIGURE 3:**
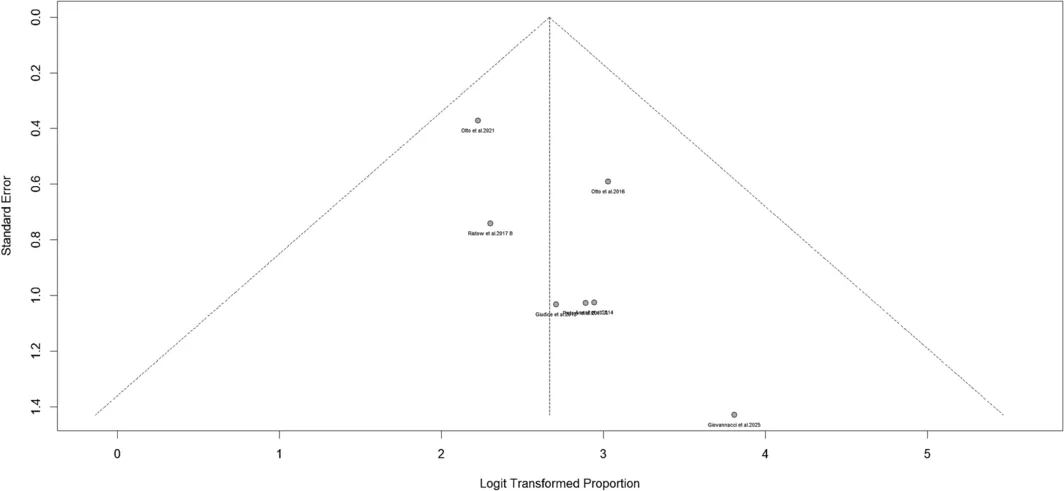
Funnel plot evaluating potential publication bias among the included studies.

## 4 Discussion

MRONJ remains a major clinical challenge, particularly among patients with oncological or osteometabolic diseases receiving antiresorptive or antiangiogenic therapies as part of their treatment regimen. Although surgical intervention represents the gold standard for MRONJ management, accurately determining the extent of necrotic bone remains a critical intraoperative task.

Several fluorescence modalities have been investigated, employing different illumination systems and detection filters to enhance the identification of necrotic bone and improve the success of surgical treatment.

In this systematic review, we observed an overall success rate of 88.5% for MRONJ lesions treated with fluorescence‐guided surgery, which was noticeably higher than the success rate achieved with conventional surgery in the included comparative studies (73.5%). These findings support the hypothesis that FGS enhances surgical precision by facilitating more accurate bone resection, thereby contributing to improved long‐term healing. However, while this difference appears clinically meaningful, it should be interpreted with caution due to the limited number of controlled trials and residual methodological heterogeneity.

Among the fluorescence modalities, AF was the most frequently employed technique. AF is based on blue excitation light within the 400–460 nm wavelength range, combined with a green filter and imaging system, allowing visualisation of the characteristic apple‐green fluorescence emitted by healthy bone tissue (Tiwari et al. 2020). This visual contrast enables surgeons to distinguish healthy from necrotic bone during the procedure, thereby improving resection precision. AF‐based surgery, as reported by Pautke et al. (2011), Aljohani et al. (2018), Otto et al. (2021) and Giovannacci et al. (2025), has consistently demonstrated success in achieving mucosal healing and reducing recurrence. In this review, AF‐based surgery achieved an overall success rate of 85.34%. These findings further support the role of AF in enhancing intraoperative visualisation and improving healing outcomes (Giovannacci et al. 2025).

TF achieved the highest cumulative healing rate (90.06%), although it requires the systemic administration of doxycycline or minocycline prior to surgery, which may not be suitable for all patients. Otto et al. (2016) demonstrated its effectiveness despite the potential systemic effects associated with preoperative doxycycline administration.

Comparative data among fluorescence systems remain limited. Ristow et al. (2017) reported no significant differences between TF and AF, whereas Giudice et al. (2018) observed improved outcomes with AF compared with conventional surgery. More recently, Zhi et al. (2025) employed ICG‐NIRF imaging, demonstrating outcomes consistent with those of other fluorescence‐assisted techniques. This method, already well established in several surgical specialties, enables real‐time perfusion assessment and deeper tissue visualisation, further supporting its potential as an adjunct in MRONJ surgery. Hwang et al. (2025) introduced a biofluorescence imaging system, a non‐invasive technique that does not require exogenous dye administration, achieving complete healing and no postoperative complications. Nevertheless, this study was excluded from the present review due to the lack of a defined clinical healing criterion. Overall, the current evidence remains insufficient to determine the superiority of one fluorescence modality over another, although TF presently demonstrates the highest proportional success rate (90.06%), followed by AF (87.05%).

In light of these findings, several methodological and clinical considerations must be addressed.

The first issue concerns the overall low quality and methodological inconsistency of the included studies. Healing in MRONJ is often slow and may require several months to stabilise; therefore, clearly defined follow‐up time points, preferably at 6 and 12 months, are essential for reliable outcome evaluation. Indeed, complete healing frequently occurs only after prolonged intervals. Hauer et al. (2020) reported complete second‐intention healing occurring over a six‐month period. Furthermore, clinical healing should ideally be corroborated by three‐dimensional radiographic assessment, as clinical closure alone may not accurately reflect true bone resolution. Most of the studies included in this review reported only clinical outcomes.

Secondly, potential differences in healing outcomes between bisphosphonate‐related and denosumab‐related MRONJ lesions are of considerable clinical interest and warrant further investigation. However, a reliable comparative analysis could not be performed in the present review. Bisphosphonate‐related MRONJ lesions were reported in all included studies, whereas denosumab‐related lesions were described in only four studies and were often pooled with bisphosphonate cases. Due to this heterogeneity and the absence of stratified outcome data, a direct comparison of healing rates between bisphosphonate‐ and denosumab‐related MRONJ lesions was not feasible. Another relevant aspect concerns outcome definitions and recurrence management. Assaf et al. (2014) classified the appearance of MRONJ at a different site as recurrence. However, the SIPMO‐SICMF guidelines clearly differentiate between recurrence, defined as the reappearance of osteonecrosis at the same site within 12 months and new localisation, which refers to lesions developing at a different site or occurring after more than 12 months. Moreover, recurrence management represents an important secondary observation. Many treatment failures required re‐operation, typically between 3 and 6 months after the initial surgery, and the success rate following re‐intervention was generally higher. However, since secondary healing was not an endpoint of the present review, re‐operated cases were considered failures at first intention.

Diagnostic criteria were also inconsistently reported, and not all studies employed CT‐based staging for surgical planning. Although fluorescence enhances intraoperative visualisation of necrotic versus vital bone, preoperative CT assessment and intraoperative evaluation of residual vital bone remain essential components of surgical decision‐making. Not all included studies considered CT imaging mandatory for treatment planning.

Finally, several technical nuances warrant consideration. First, the commonly used fluorescence devices provide qualitative, operator‐dependent visualisation rather than quantitative fluorescence assessment. For this reason, interpretation of fluorescence patterns lacks an objective and standardised threshold, and the decision to extend or terminate bone resection therefore remains dependent on the surgeon's judgement. This limitation may reduce reproducibility among operators and centres, potentially contributing to inter‐study variability. Second, fluorescence is influenced by the optical properties of tissues. While cortical bone can generate a clearer contrast, fluorescence interpretation becomes less reliable in the presence of trabecular bone, marrow cavities, blood or soft tissue. These conditions may increase the risk of ambiguous margins, potentially resulting in either excessive resection or residual necrotic bone. Thus, fluorescence alone may be misleading and should currently be regarded as an adjunctive strategy rather than a replacement for the established multimodal approach—based on the patient's medical history and clinical assessment, preoperative three‐dimensional imaging, meticulous preoperative planning and conventional intraoperative criteria (bleeding, colour and texture). Future studies should prioritise the standardisation of image acquisition protocols to evaluate the influence of variables such as ambient light conditions, camera–tissue distance, tissue type, device‐specific parameters and the development of quantitative or semi‐quantitative techniques to improve objectivity and reproducibility.

## 5 Limitations

Despite encouraging findings, the available evidence should be interpreted with caution. Several limitations emerged from the present review. First, the included studies exhibited substantial clinical heterogeneity, particularly regarding lesion site, MRONJ stage, therapeutic approach, outcome definition and duration of follow‐up. Moreover, high‐quality RCTs with large cohorts and long‐term follow‐up are lacking, and only a minority directly compared fluorescence‐guided surgery with conventional resection.

Another major limitation concerns the variability in perioperative antibiotic protocols. TF‐guided procedures most commonly involved doxycycline 100 mg twice daily for seven to 10 days, whereas other studies adopted amoxicillin‐clavulanate for 1 week before and after surgery, substituting clindamycin 600 mg three times daily in allergic patients. Alternative protocols employed minocycline 100 mg twice daily for 10 days, frequently combined with metronidazole 1 g/day from several days before surgery to 3 weeks postoperatively, alongside 0.12% chlorhexidine mouth rinses.

Similarly, follow‐up intervals varied widely, from as little as 2–4 weeks to more than 30 months, further complicating inter‐study comparability and the interpretation of true healing outcomes.

Finally, anatomical site and disease staging were inconsistently reported, and fluorescence acquisition procedures were not standardised across studies. Therefore, uniform fluorescence protocols and well‐designed controlled trials are urgently required to identify the most effective modality in relation to MRONJ stage, anatomical localisation and patient‐specific characteristics.

## 6 Conclusion

Fluorescence‐guided surgery represents a promising adjunctive technique in the surgical management of MRONJ. It may enhance intraoperative visualisation of necrotic versus vital bone, contributing to high healing rates.

However, the current evidence is limited by methodological heterogeneity and the overall low quality of the existing studies. Larger prospective randomised trials with standardised protocols, radiographic confirmation of healing and a minimum follow‐up of 12 months are required before definitive recommendations can be established.

## Author Contributions

**Paolo De Angelis:** conceptualization, investigation, methodology, data curation, writing – original draft, writing – review and editing. **Alessandro Donato Tescione:** writing – original draft, data curation, investigation, resources. **Ivan Michele Aniceto:** investigation, writing – original draft, data curation, resources. **Cosimo Rupe:** validation, visualization, formal analysis, software. **Gioele Gioco:** writing – review and editing, validation, visualization, methodology, investigation. **Romeo Patini:** methodology, formal analysis, software, validation, visualization. **Carlo Lajolo:** conceptualization, project administration, supervision, funding acquisition, visualization, validation.

## Funding

The authors have nothing to report.

## Conflicts of Interest

The authors declare no conflicts of interest.

## Supporting information


**Appendix S1:** PRISMA 2020 Checklist.
**Table S1:** Search strategy.

## Data Availability

The data that support the findings of this study are available from the corresponding author upon reasonable request.
